
JOURNAL INFORMATION
==============================
NLM Title Abbreviation: J Travel Med
Journal ID: J Travel Med
NLM Journal ID: 9434456
Journal ID: jtm

Journal of Travel Medicine

ISSN: 1195-1982
EISSN: 1708-8305

ARTICLE INFORMATION
==============================
PMCID: PMC8194836
PMID: 34050372
DOI: 10.1093/jtm/taab083
Article ID: taab083
Article version: 1
Subjects: Rapid Communication, AcademicSubjects/MED00295

Pfizer-BioNTech mRNA BNT162b2 Covid-19 vaccine protection against variants of concern after one versus two doses

https://orcid.org/0000-0003-0790-0506 Abu-Raddad Laith J PhD Infectious Disease Epidemiology Group, Weill Cornell Medicine-Qatar, Cornell University, Doha, Qatar
World Health Organization Collaborating Centre for Disease Epidemiology Analytics on HIV/AIDS, Sexually Transmitted Infections, and Viral Hepatitis, Weill Cornell Medicine–Qatar, Cornell University, Qatar Foundation – Education City, Doha, Qatar
Department of Population Health Sciences, Weill Cornell Medicine, Cornell University, New York, New York, USA

Chemaitelly Hiam MSc Infectious Disease Epidemiology Group, Weill Cornell Medicine-Qatar, Cornell University, Doha, Qatar
World Health Organization Collaborating Centre for Disease Epidemiology Analytics on HIV/AIDS, Sexually Transmitted Infections, and Viral Hepatitis, Weill Cornell Medicine–Qatar, Cornell University, Qatar Foundation – Education City, Doha, Qatar

Yassine Hadi M PhD Biomedical Research Center, Member of QU Health, Qatar University, Doha, Qatar
Department of Biomedical Science, College of Health Sciences, Member of QU Health, Qatar University, Doha, Qatar

Benslimane Fatiha M PhD Biomedical Research Center, Member of QU Health, Qatar University, Doha, Qatar
Department of Biomedical Science, College of Health Sciences, Member of QU Health, Qatar University, Doha, Qatar

Al Khatib Hebah A PhD Biomedical Research Center, Member of QU Health, Qatar University, Doha, Qatar
Department of Biomedical Science, College of Health Sciences, Member of QU Health, Qatar University, Doha, Qatar

Tang Patrick MD PhD Department of Pathology, Sidra Medicine, Doha, Qatar

Malek Joel A PhD Genomics Laboratory, Weill Cornell Medicine-Qatar, Cornell University, Doha, Qatar
Department of Genetic Medicine, Weill Cornell Medicine-Qatar, Cornell University, Doha, Qatar

Coyle Peter MD Biomedical Research Center, Member of QU Health, Qatar University, Doha, Qatar
Hamad Medical Corporation, Doha, Qatar
Wellcome-Wolfson Institute for Experimental Medicine, Queens University, Belfast, United Kingdom

Ayoub Houssein H PhD Department of Mathematics, Statistics, and Physics, Qatar University, Doha, Qatar

Al Kanaani Zaina PhD Hamad Medical Corporation, Doha, Qatar

Al Kuwari Einas MD Hamad Medical Corporation, Doha, Qatar

Jeremijenko Andrew MD Hamad Medical Corporation, Doha, Qatar

Kaleeckal Anvar Hassan MSc Hamad Medical Corporation, Doha, Qatar

Latif Ali Nizar MD Hamad Medical Corporation, Doha, Qatar

Shaik Riyazuddin Mohammad MSc Hamad Medical Corporation, Doha, Qatar

Abdul Rahim Hanan F PhD College of Health Sciences, QU Health, Qatar University, Doha, Qatar

Nasrallah Gheyath K PhD Genomics Laboratory, Weill Cornell Medicine-Qatar, Cornell University, Doha, Qatar
Department of Genetic Medicine, Weill Cornell Medicine-Qatar, Cornell University, Doha, Qatar

Al Kuwari Mohamed Ghaith MD Primary Health Care Corporation, Doha, Qatar

Al Romaihi Hamad Eid MD Ministry of Public Health, Doha, Qatar

Al-Thani Mohamed H MD Ministry of Public Health, Doha, Qatar

Al Khal Abdullatif MD Hamad Medical Corporation, Doha, Qatar

Butt Adeel A MD Department of Population Health Sciences, Weill Cornell Medicine, Cornell University, New York, New York, USA
Hamad Medical Corporation, Doha, Qatar

Bertollini Roberto MD MPH Ministry of Public Health, Doha, Qatar

To whom correspondence should be addressed. Laith J. Abu-Raddad, PhD, Infectious Disease Epidemiology Group, World Health Organization Collaborating Centre for Disease Epidemiology Analytics on HIV/AIDS, Sexually Transmitted Infections, and Viral Hepatitis, Weill Cornell Medicine - Qatar, Qatar Foundation - Education City, P.O. Box 24144, Doha, Qatar (lja2002@qatar-med.cornell.edu).
Electronic publication date: 2021 May 28
Electronic Location ID: taab083
Received 2021 May 24; Revised 2021 May 25; Accepted 2021 May 26
Copyright: © International Society of Travel Medicine 2021. Published by Oxford University Press.
Copyright year: 2021
License: This is an Open Access article distributed under the terms of the Creative Commons Attribution License (http://creativecommons.org/licenses/by/4.0/), which permits unrestricted reuse, distribution, and reproduction in any medium, provided the original work is properly cited.
License URL: https://creativecommons.org/licenses/by/4.0/

Keywords: case–control, one dose, infection, variant, vaccine, epidemiology, SARS-CoV-2
Page count: 13
edited-state: accepted-manuscript
article-lifecycle: PAP

==============================
### Pfizer-BioNTech mRNA BNT162b2 Covid-19 vaccine protection against variants of concern after one versus two doses

Laith J. Abu-Raddad, PhD[1,2,3*], Hiam Chemaitelly, MSc[1,2], Hadi M. Yassine, PhD[4,5], Fatiha M. Benslimane, PhD[4,5], Hebah A. Al Khatib, PhD[4,5], Patrick Tang, MD PhD[6], Joel A. Malek, PhD[7,8], Peter Coyle, MD[4,9,10], Houssein H. Ayoub, PhD[11], Zaina Al Kanaani, PhD[9], Einas Al Kuwari, MD[9], Andrew Jeremijenko, MD[9], Anvar Hassan Kaleeckal, MSc[9], Ali Nizar Latif, MD[9], Riyazuddin Mohammad Shaik, MSc[9], Hanan F. Abdul Rahim, PhD[12], Gheyath K. Nasrallah, PhD[7,8], Mohamed Ghaith Al Kuwari, MD[13], Hamad Eid Al Romaihi, MD[14], Mohamed H. Al-Thani, MD[14], Abdullatif Al Khal, MD[9], Adeel A. Butt, MD[3,9] and Roberto Bertollini, MD MPH[14]

© International Society of Travel Medicine 2021. Published by Oxford University Press.

UNCORRECTED MANUSCRIPT

UNCORRECTED MANUSCRIPT

[1]*Infectious Disease Epidemiology Group, Weill Cornell Medicine-Qatar, Cornell University, Doha, Qatar*
[2]*World Health Organization Collaborating Centre for Disease Epidemiology Analytics on HIV/AIDS, Sexually Transmitted Infections, and Viral Hepatitis, Weill Cornell Medicine–Qatar, Cornell University, Qatar Foundation – Education City, Doha, Qatar*
[3]*Department of Population Health Sciences, Weill Cornell Medicine, Cornell University, New York, New York, USA*
[4]*Biomedical Research Center, Member of QU Health, Qatar University, Doha, Qatar*
[5]*Department of Biomedical Science, College of Health Sciences, Member of QU Health, Qatar University, Doha, Qatar*
[6]*Department of Pathology, Sidra Medicine, Doha, Qatar*
[7]*Genomics Laboratory, Weill Cornell Medicine-Qatar, Cornell University, Doha, Qatar* [8]*Department of Genetic Medicine, Weill Cornell Medicine-Qatar, Cornell University, Doha, Qatar*
[9]*Hamad Medical Corporation, Doha, Qatar*
[10]*Wellcome-Wolfson Institute for Experimental Medicine, Queens University, Belfast, United Kingdom*
[11]*Department of Mathematics, Statistics, and Physics, Qatar University, Doha, Qatar*
[12]*College of Health Sciences, QU Health, Qatar University, Doha, Qatar*
[13]*Primary Health Care Corporation, Doha, Qatar*
[14]*Ministry of Public Health, Doha, Qatar*

UNCORRECTED MANUSCRIPT

**Word count:** Main Text: 563 words.
**Number of figures:** 1.
**Number of tables:** 1.
**Running title:** Effectiveness of BNT162b2 one vaccine dose

**Keywords:** SARS-CoV-2, epidemiology, vaccine, variant, infection, one dose, case-control.

**Corresponding Author**: Laith J. Abu-Raddad, PhD, Infectious Disease Epidemiology Group, World Health Organization Collaborating Centre for Disease Epidemiology Analytics on HIV/AIDS, Sexually Transmitted Infections, and Viral Hepatitis, Weill Cornell Medicine - Qatar, Qatar Foundation - Education City, P.O. Box 24144, Doha, Qatar (lja2002@qatar-med.cornell.edu).

UNCORRECTED MANUSCRIPT

**Key messages and recommendations**

- This population-based study documents BNT162b2 vaccine protection week-by-week after the first dose.
- 75% of protection against infection and disease is reached 15-21 days after the first dose.
- Protection increased most rapidly against hospitalization and death and slowest against B.1.351 infection.
- While protection of one dose beyond 21 days could not be assessed, findings support delaying the second vaccine dose in situations of limited vaccine supplies and high incidence.

UNCORRECTED MANUSCRIPT

Strategies for rolling out vaccination against Coronavirus Disease 2019 (Covid-19) varied across countries. A key question is whether delaying administration of the second vaccine dose to vaccinate the largest number of people in the shortest time, in situations of limited vaccine supplies and high incidence, could avert more disease cases, hospitalizations, and deaths than the current protocol of a second dose shortly after the first dose.

BNT162b2 (Pfizer-BioNTech) vaccine effectiveness against the severe acute respiratory syndrome coronavirus 2 (SARS-CoV-2) in Qatar was recently reported with focus on individuals who completed 14 days after the second dose.[1] Here, we provide a follow-up analysis of how vaccine protection develops week-by-week after the first dose.

Data for SARS-CoV-2 were extracted from Qatar's nationwide digital-health information platform. The platform hosts the national centralized SARS-CoV-2 databases that captured all vaccination records, polymerase-chain-reaction (PCR) testing, and COVID-19 hospitalizations and deaths since epidemic start.[1] The study was conducted from February 1-March 31, 2021, the period of rapid mass vaccination scale-up. Vaccine effectiveness was estimated using the test-negative case-control study design.[2] Cases and controls were matched one-to-one by age, sex, nationality, and reason for PCR testing. Effectiveness was estimated against documented infection with the B.1.1.7 or B.1.351 variants, as well as against severe, critical, or fatal disease due to any SARS-CoV-2 infection. Classification of COVID-19 case severity (acute-care hospitalizations),[3] criticality (ICU hospitalizations),[3] and fatality[4] followed the World Health Organization guidelines. Further details on study methods can be found in our previous publication.[1]

UNCORRECTED MANUSCRIPT

Between February 1-March 31, 2021, 333,764 individuals received at least one BNT162b2 vaccine dose, of whom 250,619 completed two doses. Two-thirds (60.8%) of those vaccinated were men, and the median age was 40 years. Median time elapsed between the first and second doses was 21 days, and 98.4% of individuals received their second dose ≤25 days after the first dose. Effectiveness against infection with B.1.1.7 or B.1.351 was negligible for two weeks after the first dose (Figure 1). Effectiveness increased rapidly during the third week to 65.5% (95% CI: 58.2-71.5) against B.1.1.7 and 46.5% (95% CI: 38.7-53.3) against B.1.351 (Table 1). Eventually, ≥14 days after the second dose, effectiveness reached 89.5% (95% CI: 85.9-92.3) against B.1.1.7 and 75.0% (95% CI: 70.5-78.9) against B.1.351.

Effectiveness against severe, critical, or fatal disease (predominantly due to B.1.1.7 and B.1.351[5]) was negligible during the first week, reached 26.4% (95% CI: 0.0-49.9) in the second week, and grew to 73.2% (95% CI: 56.8-84.0) in the third week (Table 1). Eventually, ≥14 days after the second dose, effectiveness reached 97.4% (95% CI: 92.2-99.5).

Development of protection against infection and disease accelerated in the third week after the first dose, right before the second dose, reaching nearly 75% of the value attained ≥14 days after the second dose. Protection increased most rapidly against hospitalization and death and slowest against B.1.351 infection. While protection of one dose beyond 21 days could not be assessed, and existing protocol requires a second dose for optimal protection, these findings support the strategy of delaying the second dose to vaccinate the largest number of people in the shortest time, in situations of limited vaccine supplies and high incidence, given the substantial protection achieved after only one dose. In areas where B.1.351 is at high incidence, delivering the second vaccine dose at 3-6 weeks after the first dose may be considered with the lower and slower build-up of protection against this variant.

UNCORRECTED MANUSCRIPT

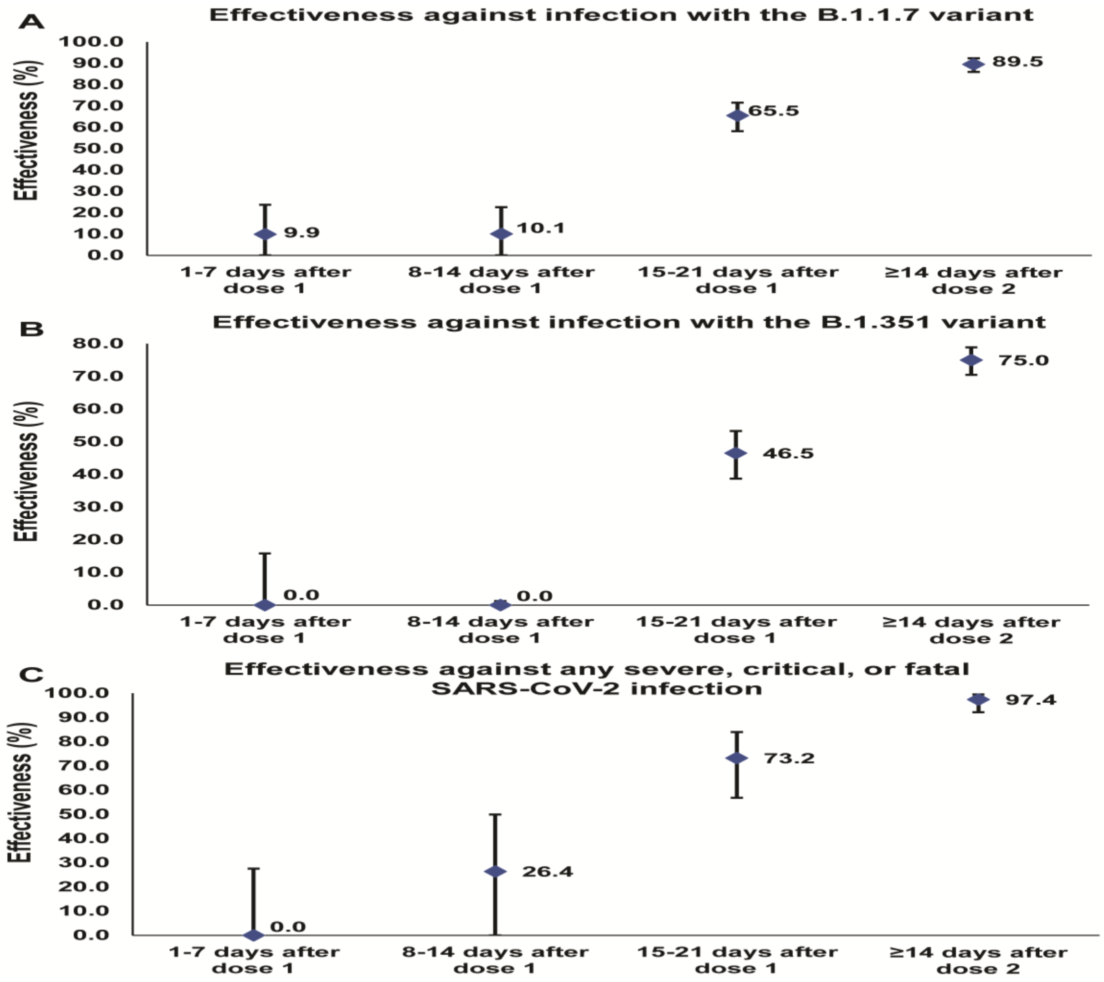

UNCORRECTED MANUSCRIPT

**Figure 1.** The messenger RNA vaccine BNT162b2 (Pfizer–BioNTech) effectiveness against infection and against disease in the weeks following the first dose. Error bars indicate the 95% confidence intervals for vaccine effectiveness estimates.

**Table 1.** The messenger RNA vaccine BNT162b2 (Pfizer–BioNTech) effectiveness against infection and against disease in the weeks following vaccination with the first dose.

| | **1-7 days after dose 1** | | | | | **8-14 days after dose 1** | | | | |
|---|---|---|---|---|---|---|---|---|---|---|
| | **Cases (PCR positive)** | | **Controls (PCR negative)** | | **Effectiveness in % (95% CI)[*]** | **Cases (PCR positive)** | | **Controls (PCR negative)** | | **Effectiveness in % (95% CI)[*]** |
| | **Vaccinated** | **Unvaccinated** | **Vaccinated** | **Unvaccinated** | | **Vaccinated** | **Unvaccinated** | **Vaccinated** | **Unvaccinated** | |
| ***Effectiveness against infection*** | | | | | | | | | | |
| Any infection with the B.1.1.7 variant[†] | 279 | 17,262 | 309 | 17,232 | 9.9 (0.0-23.7) | 346 | 17,331 | 384 | 17,293 | 10.1 (0.0-22.6) |
| Any infection with the B.1.351 variant[‡] | 276 | 19,071 | 276 | 19,071 | 0.0 (0.0-15.8) | 526 | 19,247 | 470 | 19,303 | 0.0 (0.0-1.2) |
| ***Effectiveness against disease*** | | | | | | | | | | |
| Any severe, critical, or fatal disease with the B.1.1.7 variant[§] | 12 | 431 | 19 | 424 | 37.9 (0.0-72.8) | 9 | 426 | 14 | 421 | 36.5 (0.0-76.0) |
| Any severe, critical, or fatal disease with the B.1.351 variant[¶] | 16 | 321 | 6 | 331 | 0.0 (0.0-0.0) | 17 | 322 | 6 | 333 | 0.0 (0.0-0.0) |
| Any severe, critical, or fatal disease with any SARS-CoV-2 infection[**] | 49 | 1,807 | 44 | 1,812 | 0.0 (0.0-27.6) | 52 | 1,831 | 70 | 1,813 | 26.4 (0.0-49.9) |

| | **15-21 days after dose 1** | | | | | **≥14 days after second dose** | | | | |
|---|---|---|---|---|---|---|---|---|---|---|
| | **Cases (PCR positive)** | | **Controls (PCR negative)** | | **Effectiveness in % (95% CI)[*]** | **Cases (PCR positive)** | | **Controls (PCR negative)** | | **Effectiveness in % (95% CI)[*]** |
| | **Vaccinated** | **Unvaccinated** | **Vaccinated** | **Unvaccinated** | | **Vaccinated** | **Unvaccinated** | **Vaccinated** | **Unvaccinated** | |
| ***Effectiveness against infection*** | | | | | | | | | | |
| Any infection with the B.1.1.7 variant[†] | 148 | 17,380 | 422 | 17,106 | 65.5 (58.2-71.5) | 50 | 16,354 | 465 | 15,939 | 89.5 (85.9-92.3) |
| Any infection with the B.1.351 variant[‡] | 338 | 19,400 | 623 | 19,115 | 46.5 (38.7-53.3) | 179 | 19,396 | 698 | 18,877 | 75.0 (70.5-78.9) |
| ***Effectiveness against disease*** | | | | | | | | | | |
| Any severe, critical, or fatal disease with the B.1.1.7 variant[§] | 7 | 434 | 24 | 417 | 72.0 (32.0-90.0) | 0 | 401 | 20 | 381 | 100.0 (81.7-100.0) |
| Any severe, critical, or fatal disease with the B.1.351 variant[¶] | 9 | 336 | 20 | 325 | 56.5 (0.0-82.8) | 0 | 300 | 14 | 286 | 100.0 (73.7-100.0) |
| Any severe, critical, or fatal disease with any SARS-CoV-2 infection[**] | 23 | 1,845 | 83 | 1,785 | 73.2 (56.8-84.0) | 3 | 1,692 | 109 | 1,586 | 97.4 (92.2-99.5) |

UNCORRECTED MANUSCRIPT

[*]Vaccine effectiveness was estimated using the test-negative, case-control study design.[2] Cases and controls were matched one-to-one by age, sex, nationality, and reason for polymerase chain reaction (PCR) testing. Vaccine effectiveness is given by[2] $\text{Vaccine effectiveness} = 1 - \frac{\text{vaccinated among cases} \times \text{unvaccinated among controls}}{\text{vaccinated among controls} \times \text{unvaccinated among cases}}$.

[†]Any B.1.1.7 PCR-confirmed infection. A B.1.1.7 infection is proxied as an S-gene "target failure" case using the TaqPath COVID-19 Combo Kit platform (Thermo Fisher Scientific, USA), applying the criterion of PCR cycle threshold value ≤30 for both the N and ORF1ab genes, but a negative outcome for the S-gene. The median date of vaccination was March 1 for the cases and February 28 for their matched controls.

[‡]Any B.1.351 PCR-confirmed infection. With only B.1.351 and B.1.1.7 cases identified in the viral genome sequencing after March 7, 2021, a B.1.351 infection is proxied as the complement of the B.1.1.7 criterion, that is any infection with a Ct value ≤30 for the N, ORF1ab, and S genes between March 8-31. The median date of vaccination was March 7 for the cases and March 1 for their matched controls.

[§]Any B.1.1.7 PCR-confirmed infection that led to severe, critical, or fatal disease. Severe disease, critical disease, and COVID-19 death were defined based on the World Health Organization criteria for classifying SARS-CoV-2 infection severity[3] and COVID-19-related death.[4]

[¶]Any B.1.351 PCR-confirmed infection that led to severe, critical, or fatal disease. Severe disease, critical disease, and COVID-19 death were defined based on the World Health Organization criteria for classifying SARS-CoV-2 infection severity[3] and COVID-19-related death.[4]

[**]Any PCR-confirmed infection that led to severe, critical, or fatal disease. With the dominance of both B.1.1.7 and B.1.351 variants during the study period, this effectiveness is a combined measure against both of these variants. Severe disease, critical disease, and COVID-19 death were defined based on the World Health Organization criteria for classifying SARS-CoV-2 infection severity[3] and COVID-19-related death.[4]

UNCORRECTED MANUSCRIPT

**Acknowledgements**

We acknowledge the many dedicated individuals at Hamad Medical Corporation, the Ministry of Public Health, the Primary Health Care Corporation, and the Qatar Biobank for their diligent efforts and contributions to make this study possible.

**Funding**

This work was supported by the Biomedical Research Program and the Biostatistics, Epidemiology, and Biomathematics Research Core, both at Weill Cornell Medicine-Qatar, as well as by the Ministry of Public Health and Hamad Medical Corporation. Viral genome sequencing was also supported by the Qatar Genome Programme. Statements made herein are solely the responsibility of the authors.

**Ethical approval**

This study was approved by the Hamad Medical Corporation and Weill Cornell Medicine-Qatar Institutional Review Boards with waiver of informed consent.

**Conflict of interest**

Dr. Butt has received institutional grant funding from Gilead Sciences unrelated to the work presented in this paper. Otherwise, authors declare no conflicts of interest.

**Authors' contributions**

LJA co-conceived and co-designed the study, led the statistical analyses, and co-wrote the first draft of the article. AB and RB co-conceived and co-designed the study. HC co-designed the study, performed the statistical analyses, and co-wrote the first draft of the article. All authors contributed to data collection and acquisition, database development, discussion and

UNCORRECTED MANUSCRIPT

interpretation of the results, and to the writing of the manuscript. All authors have read and approved the final manuscript.

UNCORRECTED MANUSCRIPT

**References**

1 Abu-Raddad LJ, Chemaitelly H, Butt AA, National Study Group for C-V. Effectiveness of the bnt162b2 covid-19 vaccine against the b.1.1.7 and b.1.351 variants. N Engl J Med 2021. doi: 10.1056/NEJMc2104974.

2 Jackson ML, Nelson JC. The test-negative design for estimating influenza vaccine effectiveness. Vaccine 2013; 31(17):2165-8.

3 World Health Organization. Covid-19 clinical management: Living guidance. Available from: Https://www.Who.Int/publications/i/item/who-2019-ncov-clinical-2021-1. Accessed on: May 15, 2021. 2021.

4 World Health Organization. International guidelines for certification and classification (coding) of covid-19 as cause of death. Available from: Https://www.Who.Int/classifications/icd/guidelines_cause_of_death_covid-19-20200420-en.Pdf?Ua=1. Document number: Who/hq/ddi/DNA/cat. Accessed on may 15, 2021. 2020.

5 National Project of Surveillance for Variants of Concern and Viral Genome Sequencing. Data on randomly collected samples between february 1-april 30, 2021. Data available upon request. 2021.

UNCORRECTED MANUSCRIPT
